# A pilot, case-control study on quality of life and function in adults with mild-to-moderate scoliosis treated in adolescence with physical exercises

**DOI:** 10.1186/1748-7161-7-S1-O9

**Published:** 2012-01-27

**Authors:** M Plaszewski, R Nowobilski, T Kotwicki, P Kowalski, W Chwala, M Cieslinski, R Batycki, I Cieslinski

**Affiliations:** 1 University School of Physical Education, Warsaw, Poland, Faculty of Physical Education in Biala Podlaska, Poland; 2 Department of Medicine, Jagiellonian University School of Medicine, Cracow, Poland; 3 University of Medical Sciences, Poznan, Poland; 4 Higher School of Administration, Bielsko-Biala, Poland; 5 University School of Physical Education, Cracow, Poland; 6 District Hospital, Bielsko – Biala, Poland

## Background

Studies on adults treated in adolescence surgically or with braces for idiopathic scoliosis, and on untreated subjects, indicate that both the condition and interventions can lead to psychological stress, poorer body image and self-esteem and can reduce quality of life [1-4]. Comparable evidence regarding treatment with specific physical exercises is lacking.

## Purpose of the study

We aimed to discuss the design of our ongoing case-control study on adults treated in adolescence for scoliosis with specific exercises in the Centre of Corrective and Compensatory Gymnastics in Bielsko–Biala, Poland, and to indicate tendencies shown in a pilot analysis.

## Materials and methods

Medical records of 3009 subjects who attended the Centre between 1984 and 1995 and 2158 age–matched individuals, are accessible. A pilot case-control study on 12 treated subjects and 10 controls, aged 31.4 (27 – 37) years, Cobb 35.4 (10 - 54°), was conducted. Total lung capacity and spirometry, physical activity, back pain, self-functioning, quality of life and patients’ attitudes towards treatment were measured. One-way ANOVA or a non-parametric U-test were performed.

## Results

Individual results differed, but their relation to curve angle was observed. Differences between treated and untreated individuals were ambiguous. However, inter-group analyses showed nonsignificant differences between all variables (p<0.05).

## Conclusions

Preliminary results differ from findings of the studies on braced patients: the Ste-Justine Adolescent Idiopathic Scoliosis Cohort Study, studies of Danielson and Nachemson, and continuing observations of Weinstein and coauthors. However, low power of the pilot study does not allow concluding.

## Acknowledgements

This paper is a part of a research project DS.136, University School of Physical Education, Warsaw, Poland.
